# Electrochemical Measurements of Multiwalled Carbon Nanotubes under Different Plasma Treatments

**DOI:** 10.3390/ma11101902

**Published:** 2018-10-07

**Authors:** Zulaiha Abdul Rahim, Nor Azah Yusof, Muhammad Aniq Shazni Mohammad Haniff, Faruq Mohammad, Mohd Ismahadi Syono, Nurulhaidah Daud

**Affiliations:** 1 Institute of Advanced Technology, Universiti Putra Malaysia, Serdang 43400, Malaysia; zulaiha1301@yahoo.com; 2 Chemistry Departments, Faculty of Science, Universiti Putra Malaysia, Serdang 43400, Malaysia; 3 MIMOS Berhad, Technology Park Malaysia, Kuala Lumpur 57000, Malaysia; aniqq.hanif@mimos.my (M.A.S.M.H.); matek@mimos.my (M.I.S.); nurulhaidah.daud@mimos.my (N.D.); 4 Surfactant Research Chair, Department of Chemistry, College of Science, King Saud University, P.O. Box 2455, Riyadh 11451, Saudi Arabia

**Keywords:** multiwalled carbon nanotubes, plasma treatment, Raman spectroscopy, cyclic voltammetry

## Abstract

In the present work, we described the post-treatment effects of applying different plasma atmosphere conditions on the electrochemical performances of the multiwalled carbon nanotubes (MWCNTs). For the study, a composite of MWCNTs/Co/Ti was successfully grown on the silicon substrate and then pre-treated with ammonia, oxygen and hydrogen plasma. The composite was characterized by making use of field emission scanning electron microscopy (FESEM) for the surface morphology and Raman spectroscopy for the functionalization. Further, the electrochemical measurements were performed with the use of the cyclic voltammetry (CV) applied in the 0.01 M potassium ferricyanide in 0.1 M KCl solution. On testing, the results indicated that the NH_3_-treated MWCNTs have the highest efficiency as compared to the other pretreatments and control. This increased performance of NH_3_ treated sample can be linked to the enhanced surface area of the composite, thereby improved adsorption and associated interaction with that of the analyte molecules at the electrodes. Further comparison of the electrode with that of commercial Dropsens electrodes provided the confirmation for the efficiency of the NH_3_/MWCNTs, thereby suggesting for the potentiality of applying the NH_3_ modified electrode towards electrochemical applications.

## 1. Introduction

Nanofabrication comprises the manufacturing and utilization of materials and devices with dimensions in the range of 1–100 nm and because of having diverse applications, the nanomaterials research represents an advanced developing area of nanotechnology. The nanosized materials have commanded for the better features and functions in multiple different fields including the include solar cells and electronics, energy conversion and storage, construction and engineering, biomedical, automobile, consumer goods and so forth [1]. Among many different kinds of nanomaterials in use, one of the most exciting classes is considered to be the carbon nanotubes (CNTs), or “bucky tubes”. Since their discovery in the early 1990’s by Ijima, the CNTs have inspired prevalent investigations amongst many researchers [2]. The outstanding electrical, mechanical and thermal properties offered by the CNTs made them to be potentially applicable in many different sectors of today’s technology including the solar cells, catalysis, engineering and biomedical [3]. However, the original CNTs surface reactive properties have the ability to easily form the self-aggregated nanostructures thus limit their application in the adsorption and dispersion sector. Hence, the enhancement in the properties of CNTs by means of surface modification is required so as to improve their dispersion in the organic and polymeric matrices easily [4].

The nanofabrication method making use of the plasma enhanced chemical vapor deposition (PECVD) technique called the “bottom–up” approach where the processes are elaborated for the creation of nanoscale structures and well-organized geometries. The bottom–up approach is pursued to obligate the molecular or atomic components built up into more complex nanoscale assemblies or directed self-assembly that can be found in composite technologies [5]. This technique has the ability to generate functional multi-component devices in a well-organized manner by means of controlling the self-assembling properties of the atoms and/or molecules. The PECVD technique involves the detachment of gaseous reactant molecules first, which consequently react chemically to form innumerable structures under the influence of heat, light or plasma discharge. In most cases with this technique, the solid-state stable products are formed as a result of the chemical reactions [6,7].

The various modifications onto the surface of CNTs have been developed and become commercially available in recent years such as air oxidation [8], ozone oxidation [9], wet chemical oxidation [10] and plasma treatment [11,12,13]. The employment of these treatments is to graft the functional groups onto the surface of nanotubes individually without any changes to their bulk properties. Unfortunately, the treatments involve harsh conditions leading to network destructive, damage the sidewalls and cutting the nanotubes structure which disrupt the unique properties of CNTs [14]. However, amongst these treatments, plasma modification has been the most attractive method due to its advantages such as zero pollution, shorter treatment time and the possibility to create various extra functional groups depending on the plasma atmosphere. Therefore, many efforts to understand the plasma surface treatment have been focused by the researchers [15,16,17,18,19,20].

Plasma surface treatment in general employs different gases for the study, such as oxygen, nitrogen, hydrogen and ammonia. When exposed to the plasma treatment, the excited species, radicals, electrons, ions, or UV light within the plasma interacts strongly with the surface of nanotubes leading to the breaking of sp^2^-hybridized graphite-like carbon (C=C) bonds within the CNT lattice thereby creating the defects (so-called the active sites) [20]. In this way, the functional groups occurred at the active sites can interact with the plasma-generated surface-bound radicals to form the dangling bonds [21]. Mishra et al. [22] reported the enhanced sensitivity of CNTs by increasing the oxygen content on the surface of the nanotubes through longer processing time. The modification by nitrogen plasma done by Hussain et al. [23] demonstrates the presence of pyridinic and pyrrolic functionalities on the CNTs surface leading to the improvement of reversibility of the electron transfer process. The development of plasma modifications on the surface of CNTs was studied extensively to improve the electrochemical signal by introducing various functional groups. However, to the best of our knowledge, there is no comparative study focused on different kinds of plasma atmospheres applied to the CNTs. Thus, the objective of the present study is to investigate the influence of different plasma treatments towards the surface electrochemical properties of the MWCNTs (multiwalled carbon nanotubes). For that, we first grown the MWCNTs by PECVD (plasma enhanced chemical vapor deposition) which further modified under different plasma atmospheric conditions by making use of ammonia (NH_3_), hydrogen (H_2_) and oxygen (O_2_) gases in order to create defects on the surface with an aim to enhance the electrochemical performances of the MWCNTs. 

## 2. Materials and Methods 

### 2.1. Synthesis of MWCNTs

In this study, different samples of untreated and different plasma treated MWCNTs were prepared. Each category of different treatment samples was made in triplicate. First, by making use of the magnetron sputtering technique thin film of Titanium (Ti, 10 nm thick) as the conducting layer and Cobalt (Co, 6.6 nm thick) as the catalyst for growing the CNTs were deposited onto a Si(100) substrate maintained at the reaction chamber pressure of ~10^−3^ mbar and temperature of 42 °C. The thickness of Ti and Co layer was measured in accordance with the previous work by Haniff et al. [24]. In the second step, the PECVD technique using the Oxford Instruments Nanofab-700 system maintained at ambient temperature and pressure conditions was applied for the CNTs synthesis. For that, the thin Ti-Co film formed in the first step was undergone the annealing process in vacuum at 700 °C for 2 min in hydrogen (100 sccm), plasma power of 200 W so as to form the catalyst nanoparticles (NPs). Now, 50 sccm of acetylene, C_2_H_2_ (99,99% pure) used as the precursor gas was added to the chamber and maintained the pressure of 1000 mTorr, 700 °C for 10 min to facilitate the growth of MWCNTs. The working site of MWCNTs-based electrode has the same diameter with Dropsens C101 which is 0.36 mm.

### 2.2. Post-Treatment of MWCNTs 

In the post-treatment study, each individual sample of MWCNTs was subjected to different plasma treatment so as to see the effects of surface properties with respect to the applied parameters. During the treatment, the MWCNTs were subjected to ion bombardment in the chamber which further expected to activate the surface electronic properties of the CNTs by breaking the sp^2^ (C=C) bonds [21]. The same physical plasma parameters were applied for the post treatment by only changing the types of gases, that is, NH_3_, H_2_ and O_2_ (purity of all gases is 99.99%), where we maintained a flow rate of 50 sccm, pressure of 1000 mTorr, temperature of 200 °C, radio frequency power of 20 W and for a total period of 120 s.

### 2.3. Physical Characterization

The surface morphology of all the pristine and modified CNTs was characterized by using a field emission scanning electron microscopy system (FESEM, JEOL JSM-7500F, Tokyo, Japan). The quality of the CNTs was evaluated by Raman spectroscopy (NT-MDT NTEGRA Spectra, Moscow, Russia). For the analysis, a 473 nm air-cooled laser was focused on a diffraction limited resolution of 250 nm and the samples were run for an acquisition time of 5 min.

### 2.4. Electrochemical Analysis

The electrochemical characterization of the electrodes was investigated by using potentiostatic system of cyclic voltammetry (CV). The electrochemical behavior of the Fe^2+^/Fe^3+^ redox reactions at the surface of CNTs was studied in 0.1 M KCl solution containing 0.01 M potassium ferricyanide (K_3_Fe(CN)_6_) at different scan rates in the range of 10–100 mV s^−1^. The K_3_Fe(CN)_6_ was selected as a benchmark redox system due to its surface sensitivity towards the electrochemical response, mainly for the carbon materials [24]. All the experiments were carried out at 25 °C in a typical three-electrode cell system. Commercial Metrohm Ag/AgCl electrode (3 M KCl internal solution) and carbon electrode were used as the reference and counter electrodes, respectively. The working electrode was either the bare or plasma treated MWCNTs and the geometrical area of the working electrode was set to a constant value of 0.36 cm^2^. In each electrochemical study which involves the effects of sensitivity, scan rate and NH_3_/MWCNT electrode performances were repeated three times.

### 2.5. Statistical Analysis

For the electrochemical studies, the statistical analysis was performed by using one-way analysis of variance (ANOVA) and Bonferroni’s method for multiple comparisons. 

## 3. Results and Discussion

### 3.1. Instrumental Analysis

**Figure 1:**
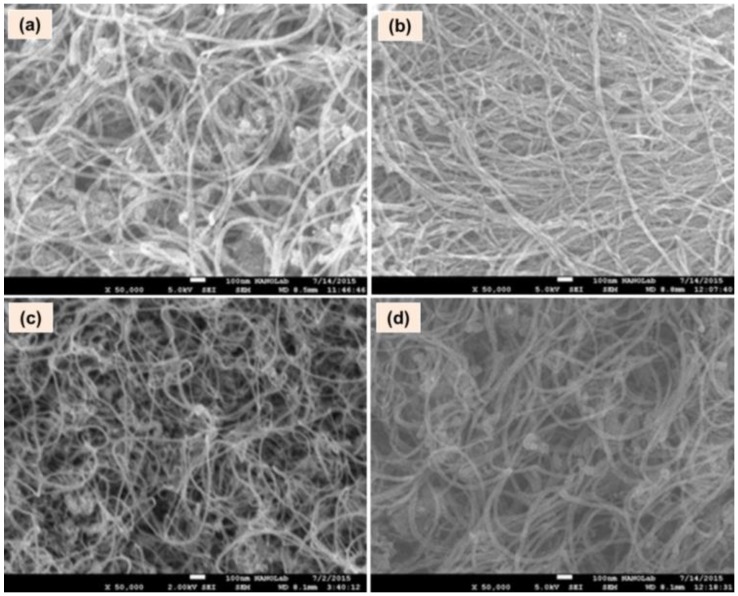
Field emission scanning electron microscopy (FESEM) images representing the surface morphology of multiwalled carbon nanotubes (MWCNTs) (**a**) before any pre-treatment and following the treatment of (**b**) NH_3_, (**c**) H_2_ and (**d**) O_2_ (resolution: ×50,000).

Figure 1a–d shows the surface morphology of MWCNTs at different stages of their plasma atmospheric pre-treatments, that is, the as-grown MWCNTs without any treatment (a), NH_3_ treated (b), H_2_ treated (c) and O_2_ treated ones (d). From the figure, it confirms for the formation of all MWCNTs in a tube like structure and in addition, there are no significant changes appearing to the morphology of samples that are being treated with plasma (Figure 1b–d). Thus, the persistence of tube like structures even after the plasma treatment confirms that there are no physical changes occurring to the morphology of the MWCNTs due to NH_3_, H_2_ or O_2_.

**Figure 2:**
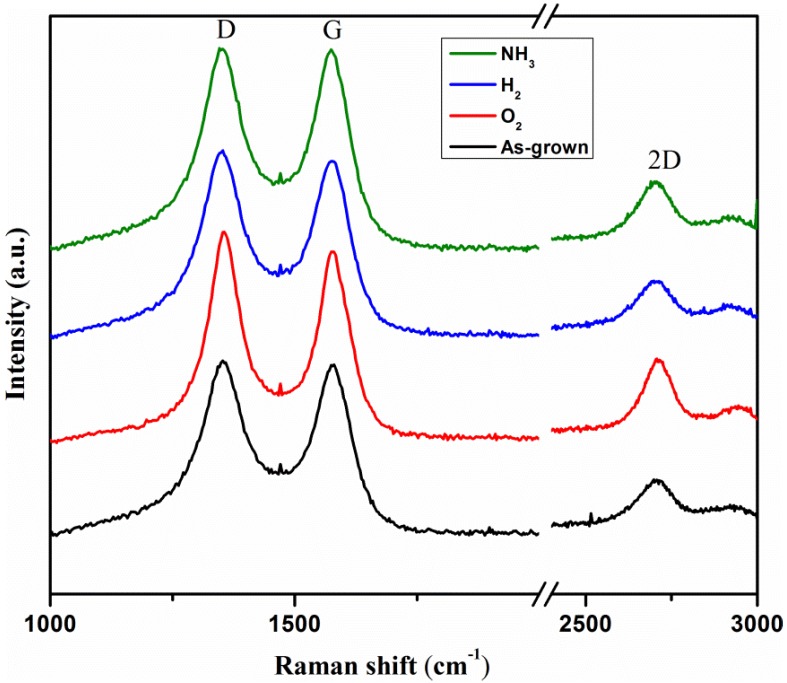
Comparison of the Raman spectra of as-grown MWCNTs with that of NH_3_, H_2_ and O_2_-plasma treated MWCNTs.

Figure 2 shows the comparison of the Raman spectrums of bare and plasma treated MWCNTs and the calculated *R* ratio values are tabulated in Table 1. From the figure, the formation of CNTs are confirmed by the appearance of three different peaks, that is, 1360 cm^−1^, 1580 cm^−1^ and 2700 cm^−1^ that can be linked to the D, G and 2D bands, respectively. The observation of both the D and 2D bands are due to the presence of defects or amorphous-phase carbon representing the plasma treatment, the modified structures of CNTs by the plasma and the G band appearance is due to the C-C stretching of the sp^2^ graphite or ordered-phase carbon. Further analysis of the ratio between D and G band intensities (*R* = I_D_/I_G_) provides the information on the relative amounts of structural defects (degree of perfection) of the nanotubes. In general, the larger ratio indicates for a reduction in the degree of perfection due to the increase of structural defects introduced by the plasma treatment [25]. The values of *R* calculated for the MWCNTs before and the plasma treatment are tabulated in Table 1. From the table, the values of *R* ratio increases for the plasma treated MWCNTs as compared against the MWCNTs without any treatment, and this is a primary indication of the formation of electron deficient moieties at the MWCNTs surface due to the plasma treatment [13]. Among all the three plasma treatments, the O_2_ treated ones has the highest defect as the value of *R* ratio observed to be 1.089, followed by NH_3_ (*R* = 1.034) and H_2_ (*R* = 1.017). The slight increase in the *R* ratio for the plasma treated MWCNTs indicate for the importance of having the necessary functionalities at the surface that are able to integrate with the lattice of nanotubes. However, the low defect ratio for the as-grown MWCNTs is due to the absence of any defective surface moieties and thereby indicating the need for the incorporation of surface groups so as to enhance the conducting properties [26].

### 3.2. Electrochemical Characterization

#### 3.2.1. Effect of Sensitivity 

**Figure 3:**
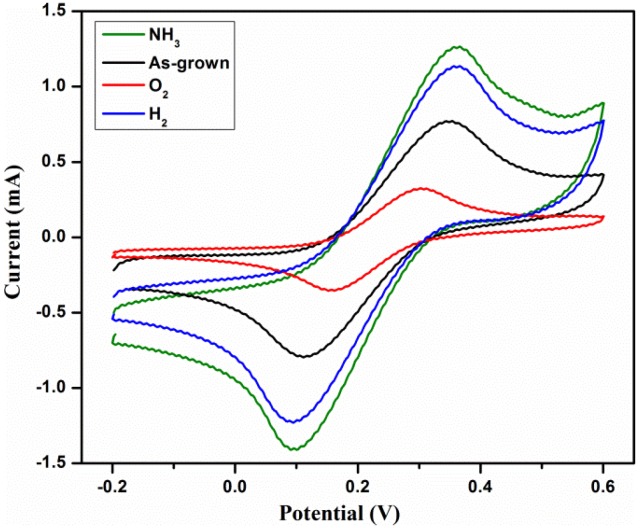
Comparison between the cyclic voltammetries (CVs) of untreated and plasma treated MWCNTs at a scan rate of 20 mV s^−1^.

The sensitivity and reversibility studies were investigated by making use of the electrochemical technique so as to observe the effect of different plasma atmosphere on the electrochemical performances of MWCNTs. Figure 3 compares the CV measurements of the bare MWCNTs, NH_3_/MWCNTs, H_2_/MWCNTs and O_2_/MWCNTs in 0.01 M ferricyanide (Fe(CN)_6_^3−/4−^) solution containing 0.1 M KCl (scan rate of 100 mV s^−1^) and the corresponding peak currents are tabulated in Table 2. Corresponding to the well-defined and quasi-reversible redox peaks shown in the CVs, the redox reactions occur directly between the electrode and electrolytes [20]. From the CV analysis shown in the Figure 3 and Table 2, the O_2_/MWCNTs seems to exhibit a lower redox peak current with reductions of 2.02 and 1.97 folds of anodic and cathodic reactions as compared to the unmodified MWCNTs. Also, there seems to be a significant enhancement in the redox peak currents for the other plasma treatments, that is, the H_2_/MWCNTs and NH_3_/MWCNTs were having increments by 1.35 and 1.5 folds for anodic peak current (i_pa_), while the cathodic peak current (i_pc_) shows the increments of 1.65 and 1.66 folds, respectively. Here, the improvement of the current signals is correlated to the increment of the diffusion rate of K_3_Fe(CN)_6_ and larger effective surface area that improved the sensitivity of the nanotubes’ surfaces [27]. Thus, from the analysis, the NH_3_ plasma treatment is said to have the best sensing characteristics as compared to the bare and other treatments.

#### 3.2.2. Effect of Scan Rate

**Figure 4:**
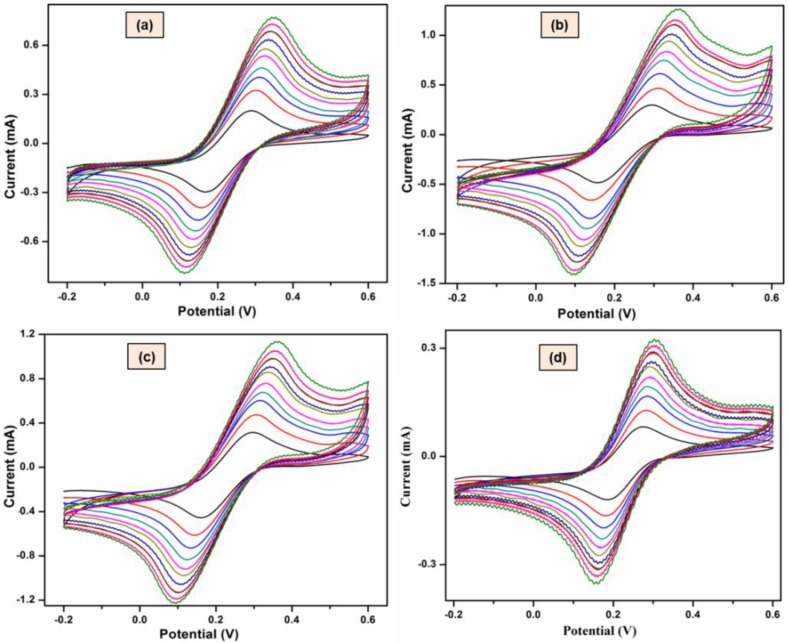
Comparison of the CVs of (**a**) untreated MWCNTs, (**b**) H_2_/MWCNTs, (**c**) NH_3_/MWCNTs and (**d**) O_2_/MWCNTs in 0.01 M [Fe(CN)_6_]^3−/4−^ at different scan rates in the range of 10–100 mVs^−1^.

**Figure 5:**
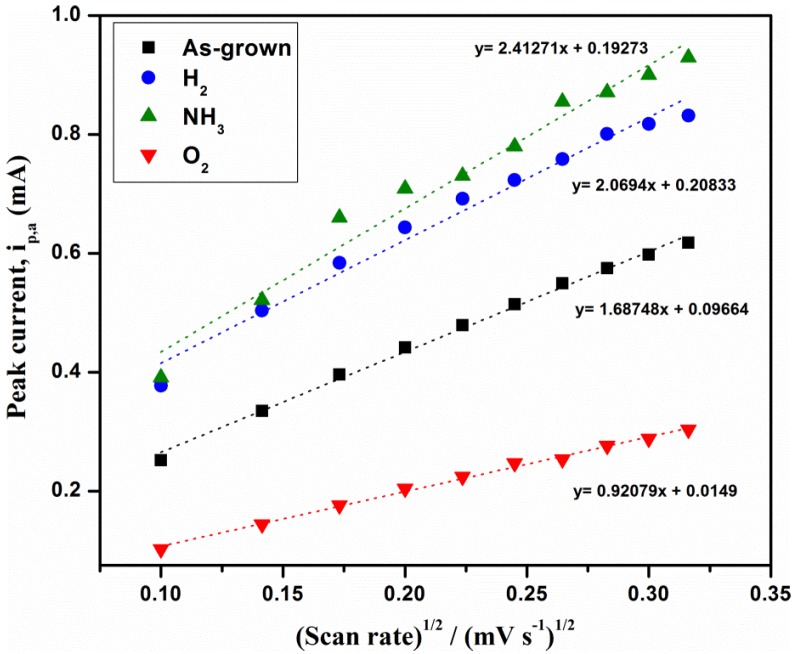
Plots of peak current against square root of scan rate of untreated and plasma-treated MWCNTs in 0.1 M KCl containing 0.01 M Fe(CN)_6_^3−/4−^ solution.

The influence of scan rates on the redox reactions for the pristine and modified MWCNTs were investigated and is shown in Figure 4. Further, the cathodic peak currents increase with an increase of potential and the corresponding peak currents (i_p_), as against the square root of scan rates (mVs^½^), are illustrated in Figure 5. From the analysis of data shown in Figure 5, the plots showed a linear relationship of i_p_ towards mVs^½^ when performed in the range of 10–100 mVs^−1^ thereby proposing a diffusion controlled process of reactants on the electrode surface. The results obtained are used to calculate the electroactive surface area (cm^2^) using Randles-Sevcik equation (1) and the values are tabulated in Table 3.

i_p_ = 2.69 × 10^5^n^3/2^AD^1/2^Cv^1/2^ (1)

where n is the number of electrons in the redox reactions, A is the electroactive surface area of the electrode (cm^2^), D is the diffusion coefficient of the molecules in solution (cm^2^/s), C is the concentration in the bulk solution (mol/mL), v is the square root of scan rate (mVs^½^) and i_p_ is the peak current (mA).

According to Equation (1), the electroactive surface area of a material is directly proportional to the peak current and thus with an increase in the surface area of the MWCNTs in our case, the peak current should be increased. From the analysis, we observed the peak current of NH_3_/MWCNTs to be the highest signal followed by H_2_/MWCNTs, bare MWCNTs and O_2_/MWCNTs. The observation of such order in the peak currents can further be linked to the electroactive surface areas of MWCNTs which is exactly same as the electrochemical response.

#### 3.2.3. NH_3_/MWCNTs Electrode Performances

**Figure 6:**
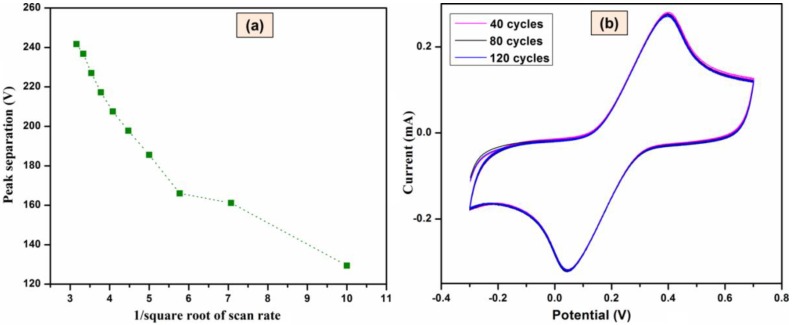
(**a**) Peak potential difference versus 1/square root of the scan rate, (**b**) multiple CVs of NH_3_/MWCNTs in 0.10 M KCl containing 0.01 M Fe(CN)_6_^3−/4−^ solution.

Further study on the activation of NH_3_ plasma alone on MWCNTs thin film was conducted. In order to govern the electron-transfer property of the species in a given environment, the peak separation of different scan rates was calculated. Figure 6a represents the data obtained from the CV studies of NH_3_/MWCNTs, where the peak potential difference versus 1/square root of the scan rate was calculated. From the figure, the peak separation is getting increased with the decrease of 1/√V/s (increased scan rates), that is, the lower scan rates are having a reversible redox couple and faster electron transfer rate. Meanwhile, the greater peak separation in higher scan rates specifies a quasi-reversible reaction. In addition, multiple cycles of CV measurements were run on NH_3_/MWCNTs sample to observe the stability of the electrode. Figure 6b shows the CVs of NH_3_/MWCNTs in 0.1 M KCl containing 0.01 M ferricyanide (Fe(CN)_6_^3−/4−^) solution that was run up to 120 cycles. From the figure, it has clearly shown for the virtual constant of either peak potential or the peak separation for the redox reactions of the ferrycyanide solution. This evidenced that the modified MWCNTs with that of NH_3_ are stable towards the detection and can be reusable for different kinds of applications.

**Figure 7:**
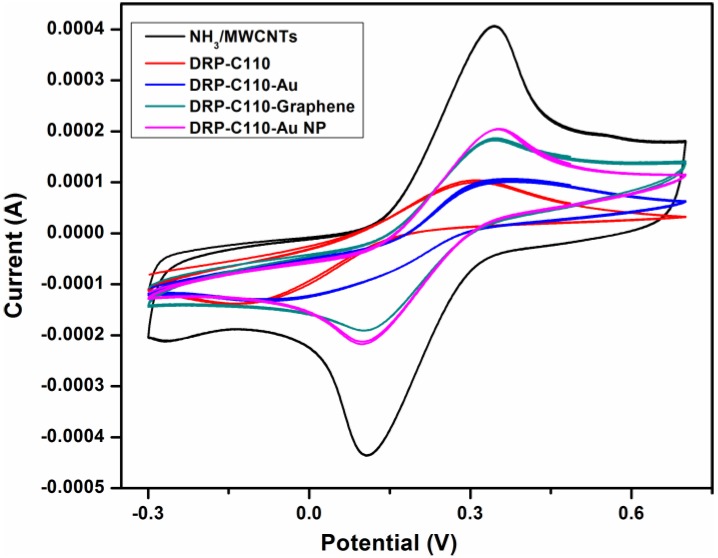
CV comparison of NH_3_/MWCNT electrodes and commercial Dropsens electrodes (DRP-C110, DRP-C110-Au, DRP-C110-graphene, DRP-C110 Au NPs).

In addition, a comparative study with that of the commercial Dropsens was carried out to inspect the sensitivity performance of the electrodes and the results are shown in Figure 7. From the figure, it can be seen clearly for the better performance of our tested electrodes in comparison to the commercial ones. The analysis of results provides the information that the bare of carbon based Dropsens-C110 shows the lowest current reading followed by Dropsens-C110-gold, Dropsens-C110-graphene, Dropsens-C110-gold NPs and our NH_3_/MWCNTs sample. In addition, we observed from the analysis that the values did not change much when we repeated the analysis for multiple times. The observation of the highest peak current for the NH_3_/MWCNTs as compared to all other samples significantly demonstrates that the plasma system upgraded the surface performance by means of enhancing the adsorption and interaction of the target analytes on the apparent of CNTs. Hence, the detection of analytes in the electrochemical system will be suggestively developed.

## 4. Conclusions

In conclusion, we proved here for the importance of having the modified surface so as to improve the electrical conductivity of the MWCNTs. For the study, the MWCNTs were successfully grown on the silicon substrate via catalytic PECVD technique followed by its characterization using the SEM and Raman spectroscopy. The modification strategies of the MWCNTs plasma treatment using different atmospheres (NH_3_, H_2_ and O_2_) proved that the ammonia plasma significantly improved the sensitivity of the electrodes up to 0.48 mA by means of increasing the effective surface area of working electrode (~0.497333 cm^2^) as compared against the bare electrode. Further, the NH_3_/MWCNTs electrode confirmed to have a better performance than the commercially available Dropsens electrodes and thereby suggesting for the potential applications of the prepared electrode towards electrochemical related studies. This improved performance of the synthesized electrode can be attributed to the increase in the surface area linked adsorption and associated electrical conductivity by means of reducing the internal resistance in the composite material. Also, the study provides information about the selection of right medium (NH_3_) as against the other two mediums (H_2_ and O_2_) for the composite where the hydrophilic and hydrophilic properties can be switched, in addition to improving the interaction with the analyte and all these factors contributes to achieve the highest efficiency of the electrode system.

## Figures and Tables

**Table 1:** Raman spectra of bare and different plasma atmosphere treated MWCNTs.

Type of MWCNTs	I_D_/I_G_ Ratio	RSD (%)	I_2D_/I_G_	RSD (%)	I_2D_/I_D_	RSD (%)
As-grown MWCNTs	0.998	2.545	0.225	2.545	0.125	4.421
NH_3_/MWCNTs	1.034	1.654	0.211	1.654	0.141	2.544
H_2_/MWCNTs	1.017	3.934	0.198	3.934	0.138	3.534
O_2_/MWCNTs	1.089	2.911	0.165	2.911	0.088	3.310

**Table 2:** Peak current and potential of bare and plasma treated MWCNTs.

MWCNTs Type	i_pa_/i_pc_	E_pa_ (mV)	E_pc_ (mV)	∆P (mV)
As grown MWCNTs	1.003	340	122	218
NH_3_/MWCNTs	1.014	347	105	242
H_2_/MWCNTs	0.974	349	100	249
O_2_/MWCNTs	1.028	298	159	139

**Table 3:** Electroactive surface areas of bare and plasma treated MWCNTs.

MWCNTs Type	i_pa_	RSD (%)	i_pc_	RSD (%)	Correlation Coefficient (*R*)	Effective Surface Area (cm²)
As-grown MWCNTs	0.335	3.612	−0.334	4.212	0.99561	0.32
NH_3_/MWCNTs	0.522	2.333	−0.536	4.213	0.99516	0.50
H_2_/MWCNTs	0.504	2.301	−0.517	3.312	0.97808	0.48
O_2_/MWCNTs	0.144	2.701	−0.140	1.521	0.9702	0.14
